# Ecological momentary assessment of momentary loneliness, body dissatisfaction, and dysregulated eating in adolescents

**DOI:** 10.1002/jcv2.70132

**Published:** 2026-05-28

**Authors:** Tyler B. Mason, Jeremy C. Morales, Kathryn E. Smith, Genevieve F. Dunton

**Affiliations:** ^1^ Department of Nutrition and Food Studies George Mason University Fairfax Virginia USA; ^2^ Department of Population and Public Health Sciences University of Southern California Los Angeles California USA; ^3^ Department of Psychiatry and Behavioral Sciences University of Southern California Los Angeles California USA

**Keywords:** adolescents, body dissatisfaction, dysregulated eating, loneliness

## Abstract

**Background:**

This objective of this study was to use ecological momentary assessment (EMA) to investigate the relationships of momentary loneliness with body dissatisfaction and dysregulated eating in a diverse sample of adolescents as well as trait peer teasing as a moderator of associations.

**Methods:**

Seventy‐four adolescents aged 13–17 completed a baseline measure of peer teasing and a 10‐day EMA protocol, reporting momentary feelings of loneliness, affect, body dissatisfaction, and eating behaviors. Mixed models were used to examine within‐ and between‐subject associations of loneliness with dysregulated eating and body dissatisfaction, including trait peer teasing as a moderator.

**Results:**

Models revealed that elevated momentary loneliness was significantly associated with higher concurrent body dissatisfaction (Estimate = 0.13, SE = 0.02, *p* < 0.001). Peer teasing moderated this relationship (Estimate = 0.12, SE = 0.03, *p* < 0.001), with stronger associations between momentary loneliness and concurrent body dissatisfaction among adolescents who experienced more teasing. Momentary loneliness was not significantly associated with subsequent dysregulated eating (LOCE: Estimate = −0.002, SE = 0.02, *p* = 0.90; Overeating: Estimate = −0.02, SE = 0.03, *p* = 0.48).

**Conclusion:**

These findings highlight the acute relationship between loneliness and greater body dissatisfaction and emphasize the importance of targeting loneliness and peer teasing in ED prevention and intervention efforts. The effect of acute loneliness on dysregulated eating may be more complex, necessitating future research clarifying this association. Preventions for EDs that focus on increasing meaningful social connection may be useful for reducing body dissatisfaction.

## Key Points

**What's known?**

- Individuals with binge‐eating spectrum disorder symptoms report higher loneliness.

**What's new?**

- Momentary loneliness was not predictive of subsequent dysregulated eating in adolescents but was associated with greater concurrent body dissatisfaction. The association between loneliness and body dissatisfaction is stronger for adolescents who report greater peer teasing.

**What's relevant?**

- Clinician's should address loneliness in youth experiencing body image concerns and/or with a history of peer teasing to prevent binge‐eating spectrum disorders.

## INTRODUCTION

Eating disorders (EDs) are characterized by harmful cognitive and behavioral patterns related to eating and body image and pose significant risks to long‐term mental and physical health (Field et al., 2012; Smink et al., 2012). Previous research has identified adolescence as a critical period for the development of EDs due to physical, psychological, and social changes that occur during this timeframe (Marzilli et al., 2018). Prevention is crucial, considering that EDs often onset in adolescence, affecting up to 17% of teenagers, particularly females (Silén & Keski‐Rahkonen, 2022). However, research on adolescent EDs is lacking, and existing models have not consistently explained their onset in youth. Body dissatisfaction and dysregulated eating (which can include binge eating, overeating, and loss of control eating [LOCE]) are key early risk factors associated with EDs (Stice et al., 2017; Stice & Shaw, 2002) and thus understanding determinants of body dissatisfaction and dysregulated eating may have positive influences on adolescent ED prevention efforts.

Eating behaviors and body dissatisfaction are dynamic and change across days but also within a day, and this is especially true among individuals with EDs (Rudiger et al., 2007; Zhu et al., 2024). Fluctuations in dysregulated eating and body dissatisfaction across the day are likely associated with various contextual, social, and psychological changes that occur in daily life. For instance, elevated negative affect and maladaptive body‐related cognitions prospectively predict increased momentary body dissatisfaction and binge eating in adults (Berg et al., 2015; Srivastava et al., 2022). Yet, social factors are under‐researched as momentary correlates of within‐day fluctuations in body dissatisfaction and dysregulated eating among adolescents.

Several reviews have highlighted the role of social factors in relation to body image and EDs (Arcelus et al., 2013; Mason et al., 2022). One factor of emerging importance is loneliness. Loneliness, or perceived social isolation, refers to a person's appraisal of the quality of social relationships (e.g., feeling alone) and differs from objective social isolation and being alone (Veazie et al., 2019). Adolescence is a time when loneliness becomes more concrete and impactful, as older children become more able to distinguish between the concepts of being alone and the experience of loneliness (Buchholz & Catton, 1999; Galanaki, 2004; Liepins & Cline, 2011). A recent systematic review of primarily cross‐sectional studies reported that loneliness was associated with higher symptom severity in EDs and greater ED symptoms in subclinical populations of adults (Rabarbari et al., 2026). Most studies have been in adults, but one study showed that greater loneliness predicted increased disordered eating across middle and high school in adolescents (Cortés‐García et al., 2022).

One's body and appearance are prominent social self‐evaluative factors in adolescence and body dissatisfaction, body image self‐conscious emotions, and social appearance anxiety are rampant during this timeframe (Bucchianeri et al., 2013; Dion et al., 2015; Sabiston et al., 2022; Zimmer‐Gembeck et al., 2018). Loneliness may contribute to ED symptoms because it is associated with attention and hypervigilance to negative and threatening stimuli as well as expectations of rejection, negative self‐evaluations, self‐disgust, and social appearance anxiety (Bangee & Qualter, 2018; Bosacki et al., 2020; Du et al., 2022; Papapanou et al., 2023; Spithoven et al., 2017; Ypsilanti, 2018; Ypsilanti et al., 2019). In addition, loneliness may curb social engagement (Zhaoyang et al., 2022), which has been shown to predict greater body dissatisfaction in daily life (Mills et al., 2014; Srivastava et al., 2022). However, research has seldom examined within‐subjects, acute associations between loneliness and body dissatisfaction and dysregulated eating in adolescents.

Additionally, peer teasing victimization is a robust risk factor for EDs, dysregulated eating, and body image concerns (Lie et al., 2019; Menzel et al., 2010). Not only is experiencing peer teasing positively associated with greater loneliness (Hayden‐Wade et al., 2005), but adolescents experiencing peer teasing may develop a cognitive bias toward attributing loneliness to appearance‐based evaluations from others. Further, adolescents with a history of experiencing teasing may internalize lack of quality friendships or social support as a reflection of their physical appearance, thereby heightening body dissatisfaction and dysregulated eating during moments of loneliness. Thus, state loneliness may be more likely to draw adolescents who have experienced teasing toward food and body image cues and thoughts, which ultimately may drive increases in body dissatisfaction and dysregulated eating. Altogether, prior findings support the idea that acute loneliness may predict subsequent body dissatisfaction and dysregulated eating in adolescents, and this effect may be stronger in youth who have experienced chronic teasing. However, these hypotheses have yet to be empirically tested.

The current study used ecological momentary assessment (EMA) to examine the role of momentary loneliness in relation to concurrent body dissatisfaction and subsequent dysregulated eating in adolescents. We hypothesized that momentary elevations in loneliness would be associated with greater concurrent levels of body dissatisfaction and predict elevated dysregulated eating at the subsequent eating episode. Loneliness overlaps with negative affect (Luo & Shao, 2023), which is a well‐defined risk factor for body dissatisfaction and EDs (Engel et al., 2016; Stice & Shaw, 2002). While some research suggests that negative affect may mediate the association between loneliness and ED‐related symptoms (Ivanova et al., 2015), extant state research using EMA in adolescents has found interpersonal/social problems have a direct effect on ED symptoms independent of negative affect (Ranzenhofer et al., 2014). However, body dissatisfaction has not been tested as an outcome. As such, it is important to control for negative affect as a potential confounder of the association between state loneliness and body dissatisfaction. The secondary goal of the study was to examine history of peer teasing as a trait moderator of the association of loneliness and body dissatisfaction and dysregulated eating (i.e., overeating and LOCE). We hypothesized that adolescents reporting higher teasing would have a stronger association between momentary loneliness and concurrent body dissatisfaction and subsequent dysregulated eating.

## METHOD

### Participants and procedures

These analyses used data from the Brain Influence on Teen's Eating (BITE) study, which was focused on understanding eating and dietary intake behaviors in a diverse community sample of adolescents across the weight spectrum. A community sample of adolescents were recruited across Los Angeles County from a variety of sources, including previous research studies, social media advertising, and community advertising. Inclusion criteria included being aged 13–17 years old and being willing to use their mobile phone or borrow a mobile phone to complete study procedures. Exclusion criteria were having contraindications for functional magnetic resonance imaging (fMRI) scans, including metal in the body that cannot be removed (e.g., braces), a history of serious head trauma, neurological disorders, or pregnant/breastfeeding.

Parents completed informed consent, and adolescents gave verbal and written assent. Interested parents or children completed a baseline screener, and potentially eligible participants were contacted via phone and email by the researchers. If the participant met the eligibility criteria, he/she was invited to participate in the study. The study was completed in two separate phases: (1) an in‐person clinic visit and (2) a 10‐day period of EMA in the participant's natural environment. At the in‐person clinic visit, participants completed informed consent/assent, anthropometric measurements, the neuroimaging protocol, and training on EMA. After leaving the clinic, participants completed the 10‐day EMA protocol and two 24‐h recalls.

EMA was completed using the Lifedata EMA application for smartphones (www.lifedatacorp.com). Adolescents used a mobile phone to complete randomly‐prompted EMA surveys on their own phone, including three prompts each weekday during non‐school time weekdays (3:30pm–9:30pm) and seven prompts each day on weekend days (9am–10pm). EMA measures were collected using (1) signal‐contingent reporting, which requires participants to complete assessments occurring at various times throughout the day in response to semi‐random prompts, and (2) event‐contingent reporting, which requires participants complete a survey whenever they ate something. For signal‐contingent reporting, semi‐random prompts were generated in stratified random sampling windows, with one prompt randomly occurring during each window. Adolescents did not complete signal‐contingent surveys on weekdays while at school to prevent classroom interruptions.

At the end of the study visit, adolescents received information and training on the EMA protocol and were given the opportunity to ask questions about the protocol. Participants were instructed not to complete entries at times when they feel they are not able or when safety is a concern (such as when driving or in class). However, they were encouraged to complete the entry as soon as possible when able and received reminder prompts if it was unanswered. Compliance was monitored, and participants with poor compliance received reminders about prompt completion. Participants received monetary compensation for participating in the study; this includes $100 for the in‐person and EMA protocol plus a $20 bonus if they had a compliance rate of 80% or higher in response to the EMA prompts.

### Measures

#### Baseline measures

##### Anthropometric measurements

Anthropometric measures were taken by a portable stadiometer and electronically calibrated digital scale. Age‐ and gender‐specific BMI z‐scores were determined using age‐based pediatric growth reference charts (Shypailo, 2020).

##### Global eating disorder symptoms

For descriptive purposes, the ED Examination Questionnaire—Adolescent version (EDEQ‐A; Carter et al., 2001) assessed global ED psychopathology. Adolescents respond to items about eating, weight, and shape concerns over the past 14 days. A composite global score was computed (ranging from 0 to 6), with higher scores indicating greater ED psychopathology.

##### Perceived peer teasing

The Perception of Teasing Scale (POTS; Thompson et al., 1995) is an 11‐item scale that assesses the frequency of weight‐related teasing and competency‐related teasing and the effects of both types of teasing. A sample item measuring weight‐related teasing is: “People made jokes about you being heavy” and a sample item measuring competency‐related teasing is: “People laughed at you because you didn't understand something.” Responses are rated on a five‐point scale with responses ranging from 1 (*never*) to 5 (*very often*). The POTS demonstrated validity and satisfactory internal consistency reliability (López‐Guimerà et al., 2012). The Cronbach's alpha in the current study was 0.88.

#### EMA measures

##### Body dissatisfaction

The State Self‐Esteem Scale was used to measure momentary body dissatisfaction. Adolescents responded to two items with how they currently felt, including “I am dissatisfied with my weight” and “I feel unattractive”. Items were rated on a scale from 1 (*not at all*) to five (*extremely*). Scores were averaged such that higher scores indicated greater body dissatisfaction. These items have been used in prior EMA work (e.g., Leahey et al., 2011; Mason et al., 2021).

##### Loneliness

Adolescents indicated how lonely they felt in the past 2 hours. The item was rated on a scale from 1 (*not at all*) to five (*extremely*). Prior EMA research has used a similar item to assess loneliness (Portingale et al., 2023).

##### Negative affect

The Positive and Negative Affect Scale‐ Short Form (PANAS‐SF; Thompson, 2007) was used to assess negative affect. Participants were asked to rate their current mood. Items (i.e., distressed, upset, shame, nervous, and afraid) were rated on a scale from 1 (*not at all*) to five (*extremely*). The PANAS‐SF has been used in prior EMA studies to assess negative affect (e.g., Mason et al., 2021).

##### Dysregulated eating

When reporting eating episodes, participants answered five questions: “While you were eating, to what extent did you feel a sense of loss of control?”; “While you were eating, to what extent did you feel that you could not stop eating once you started?”; “While you were eating, to what extent did you feel disconnected [for example, numb, zoned out, on auto‐pilot]?”; “To what extent do you feel that you overate?”; and “To what extent do you think that others would consider what you ate to be an unusual or excessive amount of food?”. The response option for each item was a scale from 1 (*not at all*) to five (*extremely*). These items have previously been administered to adolescents using EMA (Goldschmidt et al., 2022). Outcomes included overeating and LOCE, as our prior analysis showed that the five items load onto two separate within‐subject outcomes (Luo et al., 2025).

### Statistical analyses

Descriptive analyses were completed in SPSS v29.0. Sample demographics were assessed. EMA compliance to signal‐contingent prompts was computed by dividing the completed prompts by the total prompts received, and correlations and *t*‐tests were used to examine demographic factors associated with compliance. Covariates (i.e., sex at birth, age, BMI‐z, sexual identity, Hispanicity, and free or reduced lunch status) were screened and included in further analyses if related to dependent variables.

Using the glmer and lmer packages in R v4.5.0, generalized linear mixed models or linear mixed models were used to examine the association of loneliness with dysregulated eating (i.e., LOCE and overeating) and body dissatisfaction. EMA negative affect was included as a covariate in models. Loneliness and negative affect were decomposed into within‐subjects and between‐subjects components by creating group‐mean centered and grand‐mean centered predictor terms, respectively (Curran & Bauer, 2011). For dysregulated eating, a gamma outcome function was used to account for non‐linearity. Also, for dysregulated eating outcomes, the within‐subjects loneliness and negative affect terms were lagged to the subsequent nearest eating episode; effects were not lagged across days for any models. For body dissatisfaction, a linear mixed model was used given the distribution was normal. To examine peer teasing as a moderator, models were run again adding the main effect of peer teasing and the interactions of peer teasing × within‐subjects loneliness and peer teasing × between‐subjects loneliness. Significant interactions were plotted using the sjPlot package.

## RESULTS

Seventy‐five adolescents enrolled in the study, yet one participant completed <5% of EMA prompts and was removed from analyses. The participant demographic characteristics (*N* = 74) are presented in Table 1. Adolescents were on average in early‐to‐mid adolescence (Mean_age_ = 15.68 ± 1.15 years) and showed mild‐to‐moderate levels of ED concerns (Mean EDEQ‐A Global score = 2.26 ± 1.17). With regard to gender and sexual identity, most participants reported female gender identity (58.1%) and identified as straight (68.9%), with a minority of the sample reporting being another sexual identity. Regarding race and ethnicity, about 37.8% reported being Hispanic ethnicity and 60.3% reported being a non‐White race, suggesting diversity in race and ethnicity.

**TABLE 1:** Descriptive statistics for demographics (*N* = 74).

Characteristic	M(SD) or %	Range
Age	15.68 ± 1.15	13–17
BMI‐z	0.70 ± 1.07	−3.34–2.55
EDEQ global score	2.26 ± 1.17	1.00–5.79
Sex assigned at birth		
Female	62.2%	
Male	37.8%	
Gender identity		
Female	58.1%	
Male	39.2%	
I do not identify as either male or female	1.4%	
I'm not sure yet	1.4%	
Sexual identity		
Straight, not gay	68.9%	
Gay or lesbian	4.1%	
Bisexual	16.2%	
I am not sure yet	6.8%	
Something else	4.1%	
Hispanic, Latino/a or Spanish origin		
Yes	37.8%	
No	62.2%	
Race/Ethnicity		
Asian	27.4%	
Black or African American	6.8%	
White	39.8%	
Other	26%	
Grade		
8th grade	5.4%	
9th grade	14.9%	
10th grade	24.3%	
11th grade	21.6%	
12th grade	33.8%	
In free or reduced lunch program		
Yes	44.6%	
No	55.4%	

*Note*: Data are mean (SD) or %. Missing data are excluded from percentages.

Abbreviations: BMI‐z, body mass index z‐score; EDEQ, Eating Disorder Examination Questionnaire.

The mean EMA compliance was 67.48% (SD = 23.65%; Range: 10.35%–97.83%). On average, each participant completed 27.55 signaled prompts (SD = 10.62; Range:3–45). There were 2162 signal‐contingent prompts completed, and 856 event‐contingent prompts completed. Across both prompt types, there were 1388 eating episodes reported. EMA compliance was unrelated to demographic variables. Older adolescents reported higher LOCE (*B* = 0.06, *p* = 0.04), OE (*B* = 0.05, *p* = 0.05), and marginally greater body dissatisfaction (*B* = 0.13, *p* = 0.06). Adolescents with greater BMI‐z reported higher OE (*B* = 0.07, *p* = 0.02), marginally higher LOCE (*B* = 0.06, *p* = 0.06), and great body dissatisfaction (*B* = 0.25, *p* < 0.001). Female sex at birth was associated with greater body dissatisfaction (*B* = 0.50, *p* = 0.002), but unrelated to LOCE and overeating. Sexual identity, Hispanicity, and free or reduced lunch status were unrelated to LOCE, overeating, and body dissatisfaction.

The results of the models examining associations between loneliness and dysregulated eating are displayed in Table 2. There were no within‐ or between‐subjects effects for loneliness in relation to overeating and LOCE. Within‐ and between‐subjects lagged negative affect were positively associated with greater LOCE, yet only between‐subjects negative affect was positively associated with overeating. These results indicate that when negative affect was higher compared to one's average, adolescents reported subsequent greater LOCE; and adolescents who reported greater negative affect across EMA also reported higher overeating and LOCE. There were no interactions between within‐subjects loneliness and peer teasing predicting overeating or LOCE. The models including peer teasing and the peer teasing × loneliness interactions explained an additional 2% and 1% of the variance in LOCE and overeating, respectively, compared to the main effects models.

**TABLE 2:** Generalized linear models of loneliness predicting subsequent binge‐eating symptoms, with and without moderation by baseline peer teasing.

	LOCE						Overeating					
	Model 1			Model 2			Model 1			Model 2		
	Estimate	SE	*p*	Estimate	SE	*p*	Estimate	SE	*p*	Estimate	SE	*p*
Intercept	−1.03	0.35	0.004	−1.12	0.36	0.002	−0.70	0.41	0.08	−0.83	0.41	0.04
Body mass index z‐score	0.05	0.02	0.02	0.04	0.03	0.17	0.07	0.03	0.005	0.04	0.03	0.16
Age	0.05	0.02	0.04	0.05	0.02	0.02	0.03	0.03	0.25	0.04	0.03	0.11
BS negative affect	0.30	0.08	<0.001	0.32	0.08	<0.001	0.33	0.09	<0.001	0.32	0.09	0.001
WS lagged negative affect	0.06	0.02	0.02	0.05	0.02	0.02	−0.01	0.03	0.86	−0.01	0.03	0.74
BS loneliness	0.09	0.05	0.07	0.05	0.06	0.37	0.05	0.06	0.39	0.03	0.07	0.68
WS lagged loneliness	−0.002	0.02	0.90	−0.01	0.02	0.62	−0.02	0.03	0.48	−0.03	0.03	0.27
Trait peer teasing	–	–	–	−0.07	0.13	0.55	–	–	–	0.07	0.14	0.62
WS lagged loneliness × trait peer teasing	–	–	–	0.05	0.03	0.07	–	–	–	0.06	0.04	0.10
BS lagged loneliness × trait peer teasing	–	–	–	0.08	0.06	0.17	–	–	–	0.02	0.07	0.78
Model *R* ^*2*^	0.20			0.22			0.14			0.15		

*Note*: Covariates included in models were associated with outcomes at *p* < 0.10 in univariable models.

Abbreviation: LOCE, loss of control eating.

**FIGURE 1:**
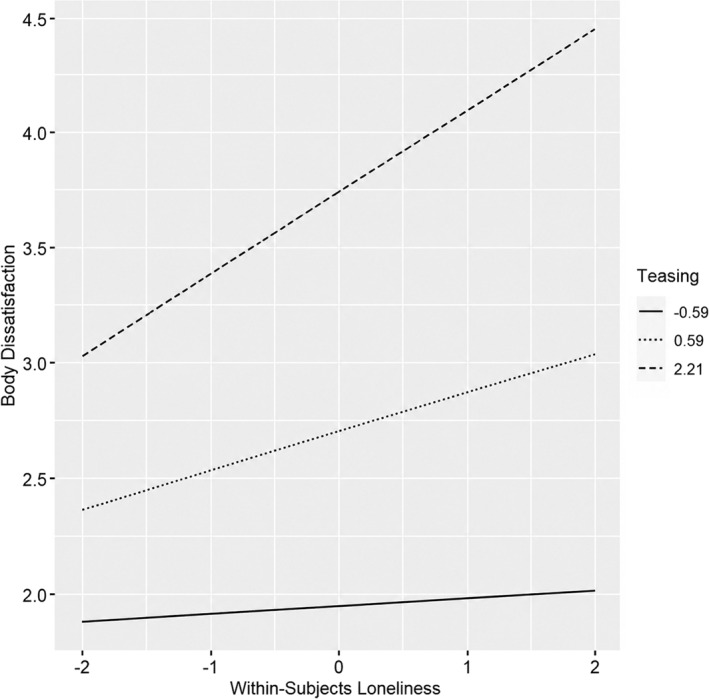
Interaction of within‐subjects loneliness and centered trait peer teasing in relation to body dissatisfaction. Teasing is plotted at −1 SD of the centered mean (−0.59), +1SD of the centered mean (+0.59), and the max value (2.21).

The results of the models examining associations between loneliness and concurrent body dissatisfaction are displayed in Table 3. In the main effects model, within‐ and between‐subjects loneliness were positively associated with greater concurrent body dissatisfaction. Therefore, when loneliness was higher compared to one's own average, then greater body dissatisfaction was reported concurrently. Also, adolescents who reported greater average loneliness across EMA also reported higher body dissatisfaction. Within‐subjects negative affect was also positively associated with body dissatisfaction such that higher negative affect than one's average was associated with greater body dissatisfaction. In the model testing the interaction effects between loneliness and peer teasing, there was a main effect for peer teasing and within‐subjects loneliness × peer teasing interaction. The interaction (see Figure 1) showed that momentary loneliness was more strongly associated with higher body dissatisfaction among adolescents with elevated levels of peer teasing. The model including peer teasing and the peer teasing × loneliness interactions explained an additional 8% of the variance in body dissatisfaction compared to the main effects model.

**TABLE 3:** Generalized linear models of loneliness and body dissatisfaction, with and without moderation by trait peer teasing.

	Body dissatisfaction					
	Model 1			Model 2		
	Estimate	SE	*p*	Estimate	SE	*p*
Intercept	−0.61	1.15	0.60	−1.13	1.06	0.29
Body mass index z‐score	0.23	0.08	0.004	0.06	0.08	0.44
Age	0.12	0.07	0.10	0.18	0.07	0.01
Female versus male sex at birth	0.43	0.17	0.01	0.52	0.16	0.002
Between‐subjects negative affect	0.21	0.21	0.31	0.19	0.20	0.33
Within‐subjects negative affect	0.18	0.03	<0.001	0.18	0.03	<0.001
Between‐subjects loneliness	0.33	0.16	0.04	0.21	0.17	0.22
Within‐subjects loneliness	0.13	0.02	<0.001	0.10	0.02	<0.001
Trait peer teasing	–	–	–	0.97	0.36	0.01
Within‐subjects loneliness × trait peer teasing	–	–	–	0.12	0.03	<0.001
Between‐subjects loneliness × trait peer teasing	–	–	–	−0.22	0.18	0.24
Model *R* ^*2*^	0.24			0.32		

*Note*: Covariates included in models were associated with outcomes in univariable models.

## DISCUSSION

The current study aimed to investigate the association between momentary loneliness and body dissatisfaction and dysregulated eating among adolescents. Additionally, we explored whether a history of experiencing peer teasing moderated these associations. Our findings extend the existing literature by demonstrating the dynamic nature of loneliness and its relationship with body dissatisfaction and dysregulated eating in adolescents' daily life, while highlighting potential interaction effects with exposure to peer teasing.

Consistent with our hypotheses, analyses revealed that both within‐ and between‐subjects loneliness were positively associated with body dissatisfaction. Adolescents reported greater body dissatisfaction at the same time as moments of heightened loneliness compared to their average levels of loneliness. This finding aligns with prior research suggesting that loneliness exacerbates self‐critical evaluations (Bangee & Qualter, 2018; Ypsilanti et al., 2019) and extends this line of work to the associations with negative body‐related cognitions; although, directionality cannot be determined with our data. The association between loneliness and body dissatisfaction was also found at the between‐subjects level, indicating that adolescents who generally felt lonelier across the study period experienced higher body dissatisfaction overall. Additionally, there was a significant within‐subjects effect for negative affect such that momentary elevations in negative affect were associated with greater body dissatisfaction. This finding suggests that loneliness and general negative affect each have independent associations with adolescents' body dissatisfaction.

Contrary to our expectations, loneliness was not significantly associated with dysregulated eating, including LOCE and overeating. This finding diverges from some prior work in youth and adults linking loneliness to disordered eating behaviors (Cortés‐García et al., 2022; Mason et al., 2016), yet other studies have also found no association between momentary loneliness and binge eating (Margaryan et al., 2025). A possible explanation for this null finding is the inclusion of negative affect as a covariate in our models. Negative affect was significantly associated with LOCE, both within‐ and between‐subjects, and with overeating at the between‐subjects level. These results suggest that the effects of loneliness on dysregulated eating may be mediated by negative affect, as proposed in prior theoretical models (e.g., interpersonal model of binge eating; Ivanova et al., 2015).

The secondary goal of this study was to examine whether self‐reported experiences of peer teasing moderated the association between within‐subjects loneliness and dysregulated eating and body dissatisfaction. For body dissatisfaction, we found that the interaction between loneliness and peer teasing was significant. Specifically, the positive association between momentary loneliness and concurrent body dissatisfaction was stronger among adolescents who reported higher levels of teasing. This finding is consistent with the notion that experiences of teasing may amplify cognitive biases toward interpreting loneliness in body‐ and appearance‐related terms (Hayden‐Wade et al., 2005). As posited, a history of peer teasing experiences may lead adolescents to attribute low‐quality friendships or limited social support to their appearance, thereby showing elevated body dissatisfaction concurrent with moments of loneliness. However, the interaction effect of loneliness and peer teasing was not significant for dysregulated eating, suggesting a more complex model of how loneliness predicts dysregulated eating in youth who vary in peer teasing experiences.

Our findings have several implications for the prevention and treatment of EDs in adolescents. The association between loneliness and body dissatisfaction highlights the potential importance of loneliness as a momentary psychosocial risk factor for EDs. Interventions and preventions that promote social connection, enhance peer support, and reduce social isolation may have downstream benefits for body image. Furthermore, the association between acute loneliness and body dissatisfaction reinforces the need for loneliness interventions in daily life, perhaps through ecological momentary interventions. Cognitive‐behavioral approaches aimed at reframing maladaptive self‐evaluations and reducing the salience of appearance‐related teasing may be particularly beneficial. The moderating effect of peer teasing demonstrates that loneliness interventions may be particularly efficacious for adolescents with a history of experiencing teasing. In addition, the effect of negative affect in predicting LOCE further bolsters the need for emotion regulation treatments for dysregulated eating prevention in youth (e.g., emotion regulation skills training; Pedrini et al., 2022).

While this study contributes to our understanding of the role of loneliness in adolescent ED prevention, several limitations should be noted. The reliance on self‐reported measures introduces the potential for bias, particularly for sensitive topics such as body dissatisfaction and dysregulated eating. Also, loneliness was measured using a single item, which may not adequately assess the multidimensional nature of loneliness, including both the emotional and social aspects of loneliness (Walsh et al., 2025). While single item measures are common in EMA loneliness research (e.g., Culbreth et al., 2021; Johnson et al., 2024), longer EMA measures have been used to assess loneliness with good within‐ and between‐subjects reliability and could be more sensitive to predicting momentary outcomes (Kuczynski et al., 2024). Additionally, our findings are based on a sample of adolescents recruited from Los Angeles County, which may limit generalizability to other populations in the United States and globally. While our sample was quite diverse, replication in diverse cultural and socioeconomic contexts is warranted. The limited between‐subject sample size may also have reduced statistical power for finding significant effects, particularly cross‐level interaction effects. Next, data were only captured during non‐school hours. Future studies should examine loneliness and eating during schooltime given that school is a unique social context for adolescents. Also, while our use of EMA captures the dynamic nature of loneliness and ED‐related symptoms, longitudinal designs across developmental transitions could provide important information on causal pathways and sensitive periods for prevention. Finally, in this study, there were six models run (i.e., two models for three different outcomes), which increases the probability of Type 1 error (Bangdiwala, 2017); although, the primary findings of the study were robust at *p* < 0.001.

This study highlights the acute and chronic associations between loneliness and body dissatisfaction, emphasizing the importance of loneliness as an independent momentary correlate of negative body image in adolescents. Further, the interaction between loneliness and peer teasing underscores the specificity of the acute loneliness‐body dissatisfaction association to adolescents with a history of peer teasing. Although loneliness was not directly linked to dysregulated eating, negative affect may be a mechanism of the association, as outlined in the interpersonal model of binge eating. Interventions targeting loneliness may be efficacious in reducing ED risk in youth by reducing state body dissatisfaction, particularly those with a history of peer teasing. However, further research is needed on the time course of the association between loneliness and body dissatisfaction.

## AUTHOR CONTRIBUTIONS

**Tyler B. Mason**: Conceptualization; methodology; investigation; formal analysis; writing—original draft; writing—review and editing. **Jeremy C. Morales**: Investigation; writing—review and editing. **Kathryn E. Smith**: Writing—review and editing. **Genevieve F. Dunton**: Writing—review and editing.

## CONFLICT OF INTEREST STATEMENT

The authors declare no conflicts of interest.

## ETHICAL CONSIDERATIONS

This study (#HS‐20‐00197) was approved by the University of Southern California institutional review board on March 05, 2020. Parents completed informed consent, and adolescents gave verbal and written assent.

## Data Availability

The data that support the findings of this study are available from the corresponding author upon reasonable request.
